# Ameliorative effect of selenium nanoparticles combined with post-conditioning on testicular ischemia–reperfusion injury in rats

**DOI:** 10.1038/s41598-026-42422-1

**Published:** 2026-03-31

**Authors:** Amena-Elmongy, Alaa Samy, Emad Tolba, Zeinab Shouman, Gamal Karrouf

**Affiliations:** 1 Department of Surgery, Anesthesiology, and Radiology, Faculty of Veterinary Medicine, Mansoura University, Mansoura, 35516 Egypt; 2 Polymers and Pigments Department, National Research Centre, 33 El Bohouth St, P.O. 12622, Dokki, Giza, Egypt; 3 Department of Cytology and Histology, Faculty of Veterinary Medicine, Mansoura University, Mansoura, 35516 Egypt; 4 Faculty of Health Science Technology, Mansoura National University, Gamasa, Egypt

**Keywords:** Testicular torsion, Ischemia reperfusion, Oxidative stress, eNOS, HSP70, Selenium nanoparticles, Post-conditioning, Biochemistry, Cell biology, Medical research, Physiology, Urology

## Abstract

Testicular torsion is a serious urological emergency that quickly cuts off blood flow to the testis, causing ischemia and subsequent reperfusion injury when blood flow restarts. This leads to oxidative stress, inflammation, and cell death, which can cause irreversible tissue damage and impairment of spermatogenesis. Although surgical detorsion is the standard treatment, post-ischemic injury remains a major obstacle to saving the testis. Selenium nano particles SeNps show strong antioxidant, anti-inflammatory, and anti-apoptotic effects, making them promising for treating ischemia–reperfusion (I/R) injury. Additionally, ischemic post-conditioning (PC) has shown protective effects in various organs, including the heart, brain, and testes, reducing reperfusion-triggered oxidative damage, lowering cell death, and improving tissue resistance to I/R injury. Investigate the synergistic effects of selenium nanoparticles and post-conditioning against testicular ischemia–reperfusion injury via testicular torsion in rats. Twenty-eight adult male rats were randomly allocated into four groups (n = 7 each): sham (SH), ischemia–reperfusion (IR), post-conditioning (PC), and selenium nanoparticles plus post-conditioning (SeNp/PC). All animals underwent right orchidectomy. In the left testis (except SH), ischemia was induced by 720° clockwise torsion of the spermatic cord for 3 h, followed by 24 h of reperfusion. In PC and SeNp/PC groups, post-conditioning was applied at the onset of reperfusion through 10 alternating cycles of reperfusion and ischemia (10 s each). Selenium nanoparticles were administered intraperitoneally at 0.5 mg/kg, 5 min before reperfusion in the SeNp/PC group. Subsequently, hormonal assays, oxidative stress biomarkers, blood profile, vascular regulation markers, apoptotic and inflammatory mediators, as well as histopathological and immunohistochemical analyses were performed. Ischemia–reperfusion (IR) injury led to increased oxidative stress markers (malondialdehyde), higher apoptotic activity (Caspase-3), and significant upregulation of inflammatory mediators (IL-6 and TNF-α). These changes were reduced by post-conditioning (PC) and were further improved with selenium nanoparticles combined with PC (SeNp/PC) compared to the SH group. Conversely, antioxidant enzymes (catalase and reduced glutathione) and reproductive hormones (testosterone, FSH, and LH) significantly increased in both PC and SeNp/PC groups relative to IR. Additionally, levels of endothelial nitric oxide synthase (eNOS), heat shock protein 70 (HSP70), and vascular endothelial growth factor (VEGF) were markedly elevated. Histological assessments (H&E and PAS staining) showed preserved testicular structure, with near-complete spermatogenesis observed in SeNp/PC-treated rats. Johnsen’s scores were 8–10, 2–3, 6–9, and 9–10 for SH, IR, PC, and SeNp/PC groups, respectively. Furthermore, SeNp/PC treatment resulted in reduced expression of pro-apoptotic Bax and inflammatory NF-κB markers. SeNp with PC boosts the alleviation of injury resulting from ischemia–reperfusion injury in the rat testis.

## Introduction

Testicular torsion is a urological emergency caused by the twisting of the spermatic cord, which obstructs blood flow to the testis and can rapidly lead to irreversible damage if not treated promptly^1^. Testicular ischemia is associated with venous congestion, arterial obstruction, edema, and necrosis, as well as impaired testicular blood flow, which leads to hypoxia and testicular dyshomeostasis, resulting in increased generation of reactive oxygen species, whereas reperfusion contributes to ischemia-primed ROS generation and increases the accumulation of intracellular calcium, oxidative stress, cellular inflammation, and apoptosis^2^. Clinically, IR injury after testicular torsion is significant because it increases the risk of long-term complications such as reduced sperm count, decreased sperm motility, and potential infertility, even if the testis is surgically salvaged^3^.

Current therapeutic strategies for testicular ischemia–reperfusion (I/R) injury primarily focus on prompt surgical detorsion to restore blood flow, but this intervention alone does not prevent further tissue damage caused by oxidative stress, inflammation, and apoptosis during reperfusion^4^. Several experimental treatments have been explored, including antioxidants (e.g., niacin, trolox, sulforaphane, and salidroside), anti-inflammatory compounds, calcium channel blockers, hormones, and newer agents such as varenicline and avanafil. These therapies have demonstrated protective benefits in animal studies by mitigating oxidative stress, suppressing inflammation, and limiting cell death^5–7^. Although promising in preclinical studies**,** the major drawback is that the majority of these treatments have yet to be confirmed in large-scale human clinical trials, and uncertainties persist regarding their optimal dosage, timing, and overall safety profiles^8^.

Nanoparticles have recently gained attention as potential therapeutic tools in testicular ischemia–reperfusion (IR) injury due to their unique physicochemical properties, targeted delivery potential, and ability to enhance the bioavailability of protective agents^9^. Research shows that they can be administered directly to testicular tissue, where they significantly reduce tissue damage by acting as antioxidants, lowering reactive oxygen species (ROS), and preventing lipid peroxidation, which helps preserve testicular structure and function^10^. Some nanoparticles possess inherent anti-inflammatory properties or can deliver anti-inflammatory drugs, suppressing pro-inflammatory cytokines (such as IL-6 and TNF-α) and downregulating inflammatory pathways, which further reduces tissue injury^11^.

Selenium nanoparticles (SeNps) are a highly bioavailable and low-toxicity form of selenium with significant antioxidant, anti-inflammatory, and therapeutic properties. Compared to traditional selenium supplements, SeNps are more efficiently absorbed, have higher biological activity, and are less toxic, making them promising for use in medicine, nutrition, and as drug carriers^12^. SeNps can enhance the activity of selenoproteins like glutathione peroxidase, which play a crucial role in maintaining redox balance and protecting cells from oxidative stress^13^. In biomedical applications, SeNps have shown potential in treating diseases related to oxidative stress and inflammation, such as cancer, diabetes, and reproductive disorders, due to their ability to scavenge reactive oxygen species and modulate immune responses^14^.

Research on selenium nanoparticles (SeNps) suggests they have strong protective effects against testicular damage, primarily due to their antioxidant properties. While there are no direct studies on SeNps in testicular torsion models, conventional selenium administration before detorsion in rats significantly reduced oxidative stress and tissue injury in both the affected and contralateral testes, as shown by lower malondialdehyde (MDA) levels, higher superoxide dismutase (SOD) activity, and improved histology^15^.

Compared with other nano-antioxidants such as cerium oxide, sodium acetate, and γ-oryzanol, selenium nanoparticles exhibit distinct mechanistic advantages. Cerium oxide nanoparticles demonstrate catalase- and superoxide dismutase–mimetic activities and can improve spermatogenic indices; however, their actions remain largely confined to direct ROS scavenging, without broader modulation of downstream signaling pathways^16^. Similarly, γ-oryzanol nanoethosomal formulations attenuate apoptosis and enhance antioxidant status, yet their influence on steroidogenic gene expression and mitochondrial integrity appears less robust than that reported for SeNps^9^.

In contrast, sodium acetate provides only minimal benefit, functioning primarily as a metabolic buffer that partially alleviates acidosis but does not influence oxidative, inflammatory, or apoptotic cascades. Consequently, it offers no substantive protection to germ, Sertoli, or Leydig cells in the context of ischemia–reperfusion injury^2^.

Ischemic postconditioning (IPostC) and remote ischemic postconditioning (RIPostC) are strategies designed to protect tissues from damage caused by ischemia–reperfusion injury. IPostC involves brief, repeated cycles of ischemia and reperfusion applied directly to the affected organ after a major ischemic event^17^. Both methods have shown promise in animal studies and early clinical trials, reducing infarct size and improving functional outcomes in conditions like heart attack and stroke, likely through mechanisms involving anti-inflammatory pathways, reduction of oxidative stress, and modulation of cell survival signaling, such as Protein Kinase B (PKB) and Extracellular signal-regulated kinases 1 and 2) ERK1/2)^18^.

### Aim of study

The present study aimed to evaluate the protective role of selenium nanoparticles in combination with ischemic post-conditioning against testicular ischemia–reperfusion injury in rats, focusing on their potential to reduce oxidative stress, inflammation, and apoptosis while preserving testicular structure and function.

## Materials and methods

### Ethical approval

The experimental protocol was approved by the Research Ethics Committee of the Faculty of Veterinary Medicine, Mansoura University, Egypt, under registration code MU-ACUC (VM.MS.23.10.90). All animal handling and experimental procedures complied with Mansoura University regulations and followed the “Guide for the Care and Use of Laboratory Animals.” The study was conducted in line with the ARRIVE guidelines.

### Animals

A total of 28 adult male Sprague–Dawley rats (mean body weight 250 ± 20 g, mean ± SD) were included in this randomized, controlled experimental study. The animals were obtained and maintained at the Medical Experimental Research Center (MERC), Faculty of Medicine, Mansoura University, Egypt, under standard laboratory conditions with a 12-h light/dark cycle, temperature of 22 ± 2 °C, and relative humidity of 65–70%. They were provided with a standard laboratory diet (prepared by the Department of Nutrition, Faculty of Veterinary Medicine, Mansoura University) and had free access to food and water. All rats were acclimatized for 7 days before the start of the experiment.

### Selenium nanoparticles

. Commercial selenium nanoparticles (GO-Bio Company, Egypt) were characterized for their physicochemical properties. Morphology and primary particle size were determined by Transmission Electron Microscopy (TEM; JEOL-JEM 1200). Hydrodynamic diameter, size distribution (polydispersity index, PDI), and zeta potential (indicating surface charge and colloidal stability).

### Study design

All rats (n = 28) underwent right orchidectomy and were then randomly assigned into four groups (n = 7 per group) based on the procedure applied to the left testis:

*SH group* only scrotal incision and gentle manipulation of the left testis, without further intervention.

*IR group* left testis subjected to 3 h of ischemia followed by 24 h of reperfusion.

*PC group* left testis exposed to 3 h of ischemia, then treated with post-conditioning (PC), followed by 24 h of reperfusion.

*SeNp/PC group* rats received 0.5 mg/kg selenium nanoparticles administered 5 min before post-conditioning, after 3 h of ischemia, and followed by 24 h of reperfusion.

### Experimental procedure

Under complete aseptic condition and general anesthesia protocol experimental surgery was induced by 10 mg/kg xylazine (XYLAJECT, ADWIA, EGYPT) and 80 mg/kg ketamine (KETAMAX, INDIA), both were given intraperitoneally, all animals were conducted to right unilateral orchidectomy while in left testes of all except SH rats were applied to scrotal incision and exteriorization of the testes then twisting the spermatic cord 720° clockwise subsequently was fixed with scrotal skin using 4–0 proline (M-NATUREⓇ, International Sutures Manufacturing co, EGYPT) 3 h for ischemia afterward repositioning to normal anatomical testicular position 24 h later representing reperfusion, while post-conditioning technique was applied only in PC and SeNp/PC immediately at reperfusion in a manner of 10 cycles of reperfusion and ischemia 10 s each while selenium nanoparticle was administered 0.5 mg/kg IP five minutes before reperfusion according to Asadpour et al.^19^.

### Euthanasia

After 24 h of reperfusion, which was performed by 120 mg/kg, i.p of Thiopental Na (THIOPENTAL INJECTION BP 2019, EPICO, EGYPT)^20^.

### Blood samples

Blood was Collected Via Cardiac Puncture. Two separate blood samples were collected; The first sample was collected in Eppendorf tubes containing K_2_EDTA (0.5 mg/ml blood) and well mixed for a complete blood picture. The second one was collected without an anticoagulant, then centrifuged at 1500 rpm for 10 min, and sera were collected and cooled at − 20 °C for serum reproductive analysis.

### Testicular tissue samples

Tissues were divided into three pieces and manipulated for different measurement purposes; The first part (0.5 g) was crushed in 5 ml ice-cold Phosphate-Buffered Saline). PBS ((PH 7.5) and centrifuged at 2000 rpm for 15 min at 4 °C, supernatant was carefully aspirated and frozen at − 20 °C for estimation of MDA, CAT, GSH, Caspase3, IL 6, TNF α, eNOS, VEGF, and HSP70. The last part was fixed using 10% neutral buffered formalin for histopathological and immunohistochemical assays.

### Laboratory examination

#### Hormonal analysis

Follicle‐stimulating hormone (FSH), luteinizing hormone (LH) (DCM009-11), and testosterone (DCMOO2-9) concentrations were quantified using a sandwich enzyme‐linked immunosorbent assay (ELISA) with assay kits from DIAMETRA (Italy), following the supplied protocols.

#### Oxidative stress and antioxidant markers

Commercially available Bio-diagnostic ready kits were acquired to evaluate malondialdehyde (MDA) (LIPID PEROXIDE, cat. no. MD 25 29), catalase (CAT) (CATALASE ASSAY, cat. no. CA 25 17) activities, and Glutathione (GSH) (GLUTATHIONE REDUCED, cat. no. GR 25 11) by an enzymatic colorimetric approach.

#### Complete blood picture

Blood profile parameters, including HB, HCT(PCV), MCV, MCH, and MCHC, were measured via Mindray animal care (BC-20 Vet).

#### Vascular regulatory protein expressions

Rat NOS3/eNOS (Nitric Oxide Synthase 3, Endothelial) ELISA Kit, cat.no. RTFI00087 and Rat VEGF ELISA Kit PicoKine® cat.no. EK0540.

#### Apoptotic and cellular stress markers

Rat Caspase 3 (Casp-3) ELISA Kit and Rat Heat Shock Protein 70 (hSP-70) ELISA Kit, cat.no. CSB-E08308r.

#### Inflammatory and immune response markers

Rat TNF-α ELISA Kit and IL-6 immunoassay Quantikine® ELISA cat.no. R6000B.

## Histopathological and immunohistochemical examination

### Sample processing and tissue preparation

Histopathological evaluation was performed by a qualified histologist who was blinded to the experimental group allocation to avoid bias. Testis samples (n = 7/Group) were fixed using 10% neutral buffered formalin, processed using ascending grades of ethyl alcohol (50%, 70%, 90%, 100%) followed by three changes of xylene, and blocks were produced after samples immersion in melted paraffin wax (3X). The produced blocks were cut using a rotary microtome at 3 µm thickness and were picked on glass slides to be stained^21^.

#### Routine staining and periodic acid-Schiff staining (PAS)

To examine tissue architecture, sections were stained with hematoxylin and eosin (HE). Testis sections were initially deparaffinized using xylene and then rehydrated through a graded ethanol series (100%, 95%, 90%, 80%, and 70%, then stained with hematoxylin for 10 min. After rinsing, they were stained with eosin for 3 min, followed by washing and dehydration^22^.

On the other hand, PAS staining was carried out for staging and Johnsen`s score detection. All slides were deparaffinized and rehydrated. Following rehydration, the slides were immersed in 0.5% periodic acid solution (Chongqin Biospes Co, CAT NO. BCS1607) for 10 min, rinsed under running tap water, and then washed again with distilled water. Next, the slides were stained with Schiff’s reagent (Chongqin Biospes Co, CAT NO. BCS1607) for 15–20 min. After staining, they were rinsed with a sulfurous acid solution composed of 10% sodium bisulfite (NaHSO₃), 1N hydrochloric acid (HCl), and distilled water. Slides were then rinsed again with distilled water and counterstained with Meyer’s hematoxylin (Chongqin Biospes Co, CAT NO. BCS1607), followed by rinsing with running tap water and distilled water. Finally, the slides were dehydrated, cleared, and mounted using Canada balsam, then covered with coverslips^23^.

#### Immunohistochemical staining

Paraffin-embedded testis sections were first deparaffinized and rehydrated. To block endogenous peroxidase activity, the sections were treated with 0.3% hydrogen peroxide (H_2_O_2_) for 10 min. After rinsing with phosphate-buffered saline (PBS, pH 7.4), the sections were incubated with 5% bovine serum albumin (BSA) in PBS for 20 min to prevent nonspecific binding. They were then incubated overnight at 4 °C with either monoclonal rabbit anti-NF-κB antibody (1:100 dilution, A2547) or monoclonal rabbit anti-Bax antibody (1: 450 dilution, GB114122).

After another PBS rinse, the sections were incubated with an HRP-conjugated goat anti-rabbit secondary antibody (1:1000; catalog no. AS014) at 37 °C for 30 min and washed again with PBS. Immunoreactivity was visualized using 3,3′-diaminobenzidine (DAB, Sigma-Aldrich, D12384) as a chromogenic substrate. Sections were counterstained with hematoxylin, dehydrated, and mounted using Canada balsam. Positive immunoreactivity appeared as brown staining^24^.

The expression of NF-κB antibody and Bax proteins was analyzed in 100 randomly selected seminiferous tubule sections from each rat^25,26^.

#### Morphometric analysis

Certain parameters were measured after examination of the HE stained sections, including seminiferous tubule diameter (SD), seminiferous epithelium thickness (ET), and luminal area width (LW)^27^. The images were captured and analyzed by ImageJ software.

Besides, at least 10 seminiferous tubules from each one of the examined groups were assessed and scored from 1 to 10 according to the severity of tubular injury^28^.

#### Statistical analysis

A one-way analysis of variance (ANOVA) was employed to compare the differences among the experimental groups. All quantitative data were expressed as mean ± standard deviation (SD). Statistical analyses were conducted using GraphPad Prism software (version 8.4.3; GraphPad Software, San Diego, CA, USA) and SPSS software (version 22; IBM Corp., Armonk, NY, USA). Tukey’s post hoc multiple-comparison test was applied to determine pairwise differences between groups. A p-value < 0.05 was considered statistically significant throughout the analysis.

## Result

### Selenium nanoparticles physicochemical features

**Fig. 1:**
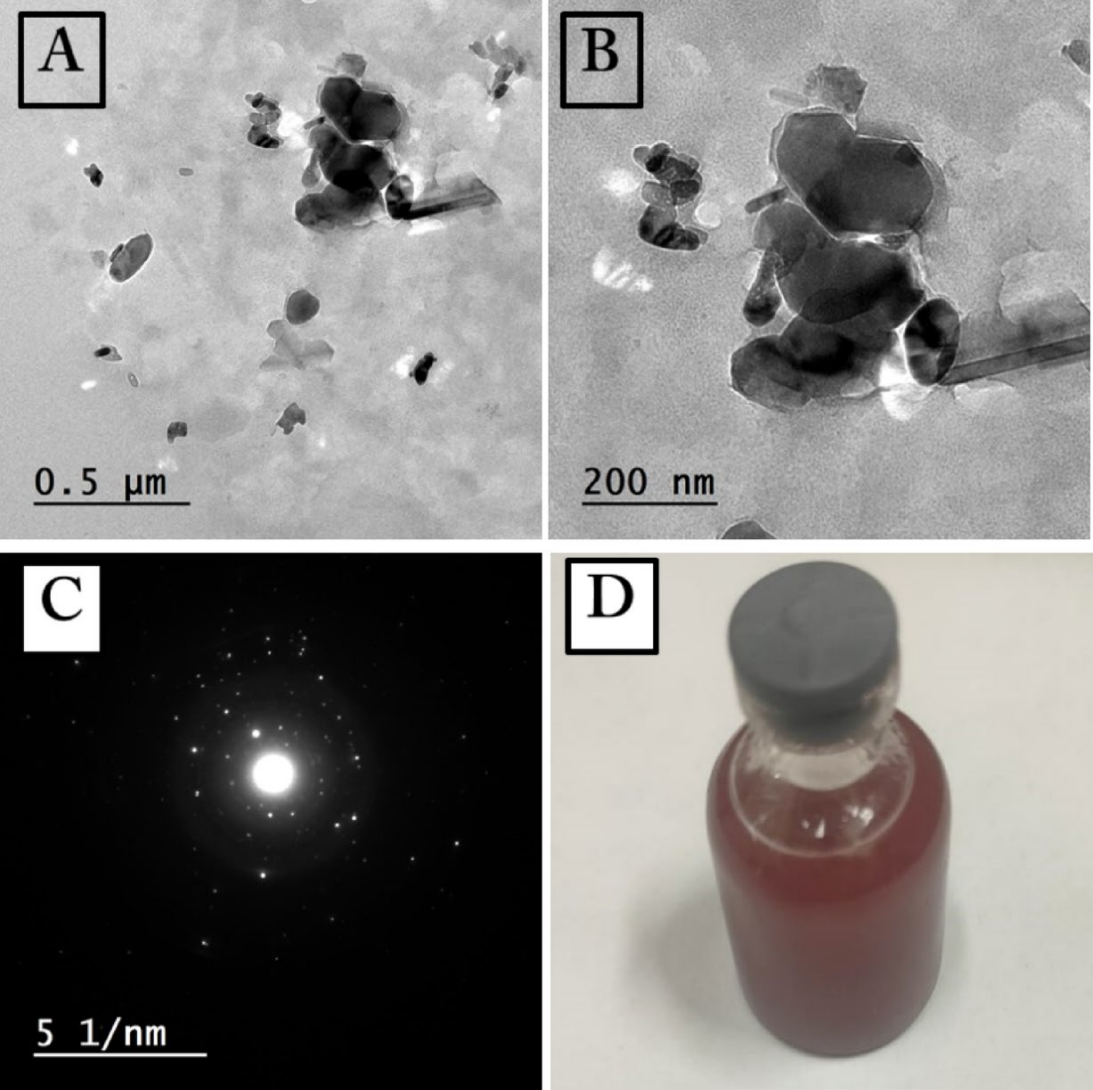
(**A** & **B**) Transmission electron microscopy (TEM) images of the SeNps (scale bar = 500 and 200 nm), (**C**) Selected diffraction of the SeNps. (**D**) Photo of SeNps colloidal solution stabilized with a 0.5% (w/v) CMC solution.

The comprehensive physicochemical features of the SeNps using TEM and DLS reveal critical insights into their morphology, colloidal stability, and hydrodynamic behavior, which are paramount for predicting their performance in subsequent biomedical applications^29^. As shown in Fig. 1a and b, TEM analysis revealed rod-shaped particles with average dimensions of 217 ± 87 nm in length and 92 ± 26 nm in width. The well-defined spots in the SAED pattern (Fig. 1) indicate the single-crystalline character of the synthesized SeNps. The polydispersity was corroborated by DLS, which yielded a high PDI of 0.61. The notable discrepancy between the TEM core size and the DLS hydrodynamic radius (511.3 nm) strongly implies that the primary nanorods form aggregates in aqueous suspension^30^ .

The positive zeta potential of + 23.71 mV indicates moderate electrostatic repulsion to prevent immediate precipitation, though the large standard deviation of ± 8.2 mV further underscores the sample heterogeneity^31^. This moderate stability underscores the need for careful handling and storage, as changes in pH or ionic strength could easily destabilize the suspension. The SeNps were stabilized in a 0.5% (w/v) sodium carboxymethyl cellulose (CMC) solution (Fig. 1d) and stored at 4 °C in the dark to prevent aggregation and photodegradation throughout the study.

### Significant modulation of reproductive hormones following treatment

**Fig. 2:**
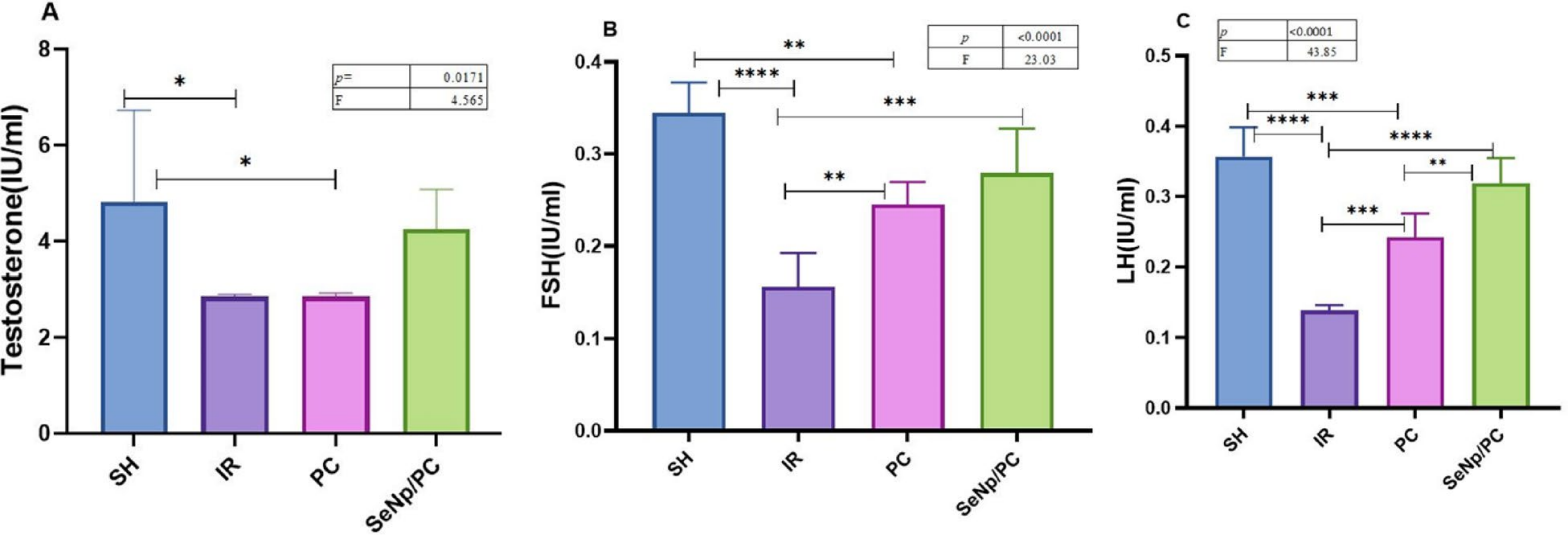
Serum levels of testosterone (**A**), follicle-stimulating hormone (FSH; **B**), and luteinizing hormone (LH; **C**) were measured 24 h post-reperfusion in the four groups. Data are expressed as mean ± SD (n = 7/group). Statistical significance was determined by one-way ANOVA followed by Tukey’s post hoc test, *p < 0.05, **p < 0.01, ***p < 0.001, ****p < 0.0001.

Serum testosterone, FSH, and LH levels showed marked alterations among the experimental groups (Fig. 2A–C) (Table 1). The I/R group exhibited a significant decline in serum testosterone (*p* = 0.0171), FSH (*p* < 0.0001), and LH (*p* < 0.0001) compared with the SH group. PC alone partially improved these hormonal levels, whereas the combined SeNp/PC treatment produced a more pronounced elevation in FSH and LH levels (*p* < 0.01–0.001) and partially restored testosterone compared with the I/R and PC groups.

**Table 1:** Serum concentrations of (a) testosterone, (b) follicle-stimulating hormone (FSH), and (c) luteinizing hormone (LH) in rats from different experimental groups. Values are presented as mean ± standard deviation (SD). Statistical analysis was performed using one-way ANOVA followed by Tukey’s post hoc test; differences were considered significant at *p* < 0.05.

Variables	Groups				F test	*P* value
	SH	IR	PC	SeNp/PC		
Testosterone	4.8 ± 1.9	2.85 ± 0.05	2.852 ± 0.07	4.252 ± 0.8	4.565	= 0.017
FSH	0.34 ± 0.03	0.15 ± 0.03	0.24 ± 0.02	0.27 ± 0.04	23.03	< 0.0001
LH	0.35 ± 0.04	0.13 ± 0.01	0.24 ± 0.03	0.31 ± 0.03	43.58	< 0.0001

Data are expressed as mean ± SD (n = 7/group). One-way ANOVA followed by Tukey’s post hoc test was used for statistical analysis. Statistical significance is considered when *p* < 0.05.

### Changes in antioxidant status and oxidative stress biomarkers

**Fig. 3:**
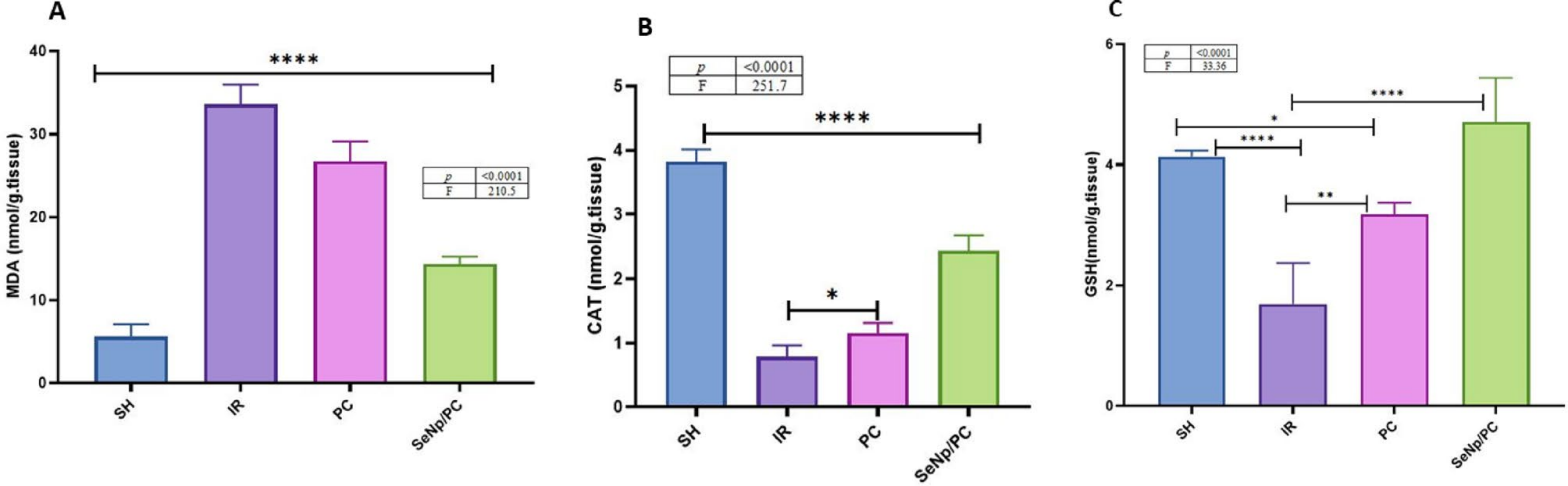
Levels of malondialdehyde (MDA; **A**), catalase (CAT; **B**), and reduced glutathione (GSH; **C**). Data are expressed as mean ± SD (n = 7/group). Statistical significance was determined by one-way ANOVA followed by Tukey’s post hoc test: *p < 0.05, **p < 0.01, ***p < 0.001, ****p < 0.0001.

Oxidative stress and antioxidants were presented in (Fig. 3A–C) (Table 2). MDA level was markedly increased in IR than in the other groups. Both CAT and GSH were reduced in IR compared with SH, SeNp/PC, and PC groups. Meanwhile, there was a significant enhancement in SeNp/PC than in PC. nevertheless, slightly reduced from SH. There was a considerable improvement in SeNp/PC than PC when compared to SH, p < 0.0001.

**Table 2:** Tissue concentrations of (a) malondialdehyde (MDA), (b) catalase (CAT), and (c) glutathione (GSH) in rats from different experimental groups. Values are presented as mean ± standard deviation (SD) for each group. Statistical significance was assessed using one-way ANOVA followed by Tukey’s post hoc test; differences were considered significant at *p* < 0.05.

Variables	Groups				F test	*P* value
	SH	IR	PC	SeNp/PC		
MDA	5.59 ± 1.50	33.56 ± 2.4	26.7 ± 2.4	14.35 ± 0.9	210.5	< 0.0001
CAT	3.82 ± 0.19	0.78 ± 0.1	1.150 ± 0.1	2.434 ± 0.2	251.7	< 0.0001
GSH	4.14 ± 0.1	1.69 ± 0.6	3.178 ± 0.1	4.708 ± 0.7	33	< 0.0001

Data are expressed as mean ± SD (n = 7/group). One-way ANOVA followed by Tukey’s post hoc test was used for statistical analysis. statistical significance is considered when *p* < 0.05.

### Variable alterations in complete blood count and hematological indices

**Fig. 4:**
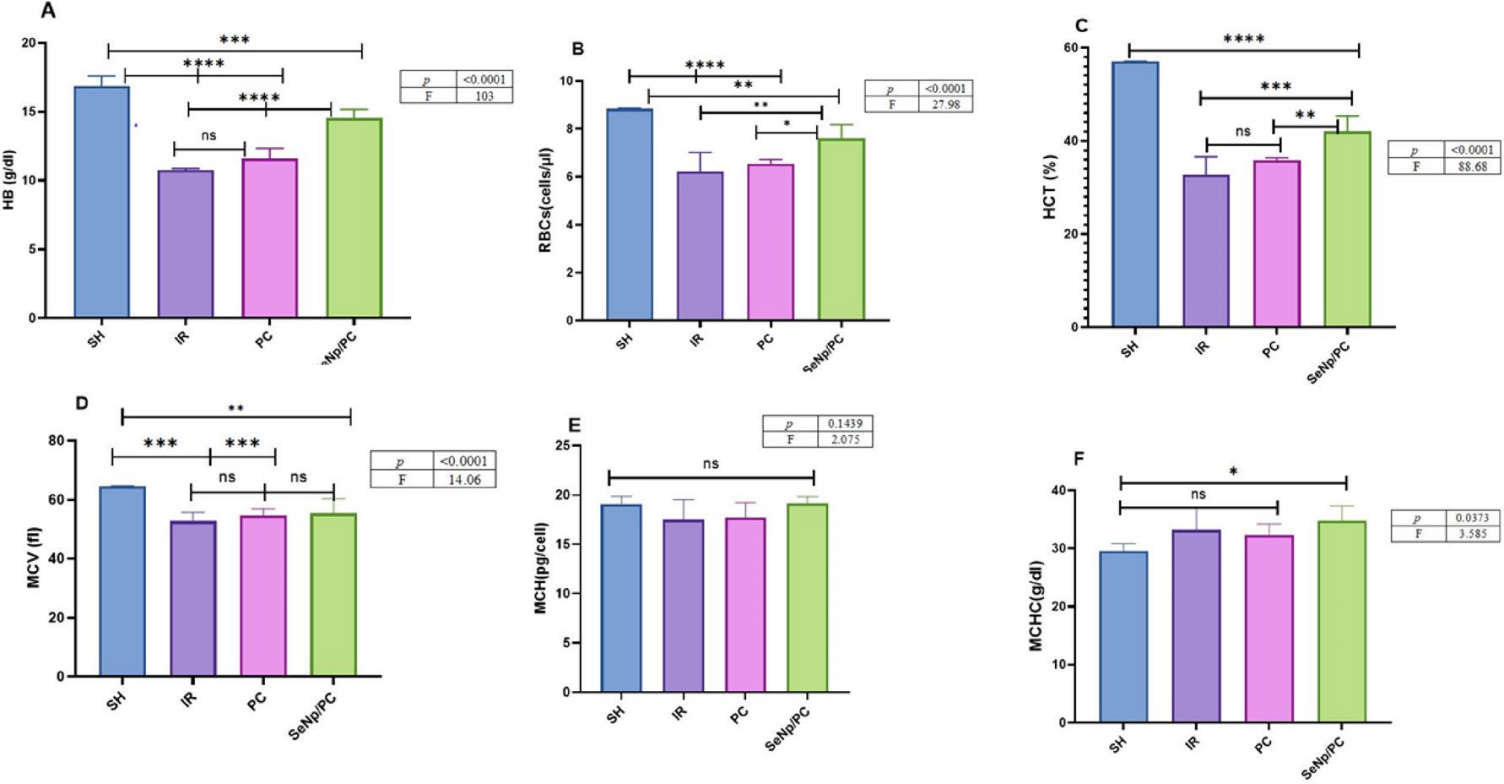
(**A**–**F**) shows Complete blood profile parameters across the study groups. No significant differences were detected between the IR and PC groups, although both showed reductions compared to SH. The SeNp/PC group demonstrated a significant elevation in Hb (*p* < 0.0001), RBCs (*p* < 0.0001), and MCV (*p* < 0.0001) relative to PC and SH. No marked changes were observed among the groups in HCT (*p* < 0.0001), MCH (*p* = 0.1439), or MCHC (*p* = 0.0373).

Blood profile parameters, as mentioned in (Fig. 4A–F) (Table 3) showed that there was no significant difference between IR and PC, while reduced than SH, as well as there was a significant elevation in SeNp/PC than PC compared with SH in HB(p<0.0001), RBCs(p<0.0001), and MCV(p<0.0001), but there was no clear variation in other parameters between study groups HCT (p<0.0001), MCH(p<0.1439), MCHC(p<0.0373).

**Table 3:** Blood concentrations of (a) hemoglobin (Hb), (b) red blood cells (RBCs), (c) hematocrit (HCT), (d) mean corpuscular volume (MCV), (e) mean corpuscular hemoglobin (MCH), and (f) mean corpuscular hemoglobin concentration (MCHC) in rats from different study groups. Values are presented as mean ± standard deviation (SD) for each group. Statistical significance was determined using one-way ANOVA, Tukey post hoc test, and GraphPad; differences were considered significant at *p* < 0.05.

Variables	Groups				F test	*P* value
	SH	IR	PC	SeNp/PC		
HB	16.86 ± 0.70	10.76 ± 0.1	11.76 ± 0.70	14.54 ± 0.60	103	< 0.0001
RBCs	8.83 ± 0.04	6.224 ± 0.7	6.554 ± 0.10	7.61 ± 0.50	27.98	< 0.0001
HCT	57.0 ± 0.10	32.74 ± 3.8	35.86 ± 0.50	42.02 ± 3.20	88.68	< 0.0001
MCV	64.51 ± 0.20	52.71 ± 3.1	54.76 ± 2.10	55.41 ± 4.90	14.06	< 0.0001
MCH	19.08 ± 0.70	17.49 ± 2.00	17.72 ± 1.40	19.15 ± 0.60	2.075	< 0.0001
MCHC	29.58 ± 1.20	33.22 ± 3.7	32.34 ± 1.80	34.73 ± 2.60	3.585	< 0.0001

Data are expressed as mean ± SD (n = 7/group). One-way ANOVA followed by Tukey’s post hoc test was used for statistical analysis. Statistical significance is considered when *p* < 0.05.

### Vascular regulatory protein expressions

**Fig. 5:**
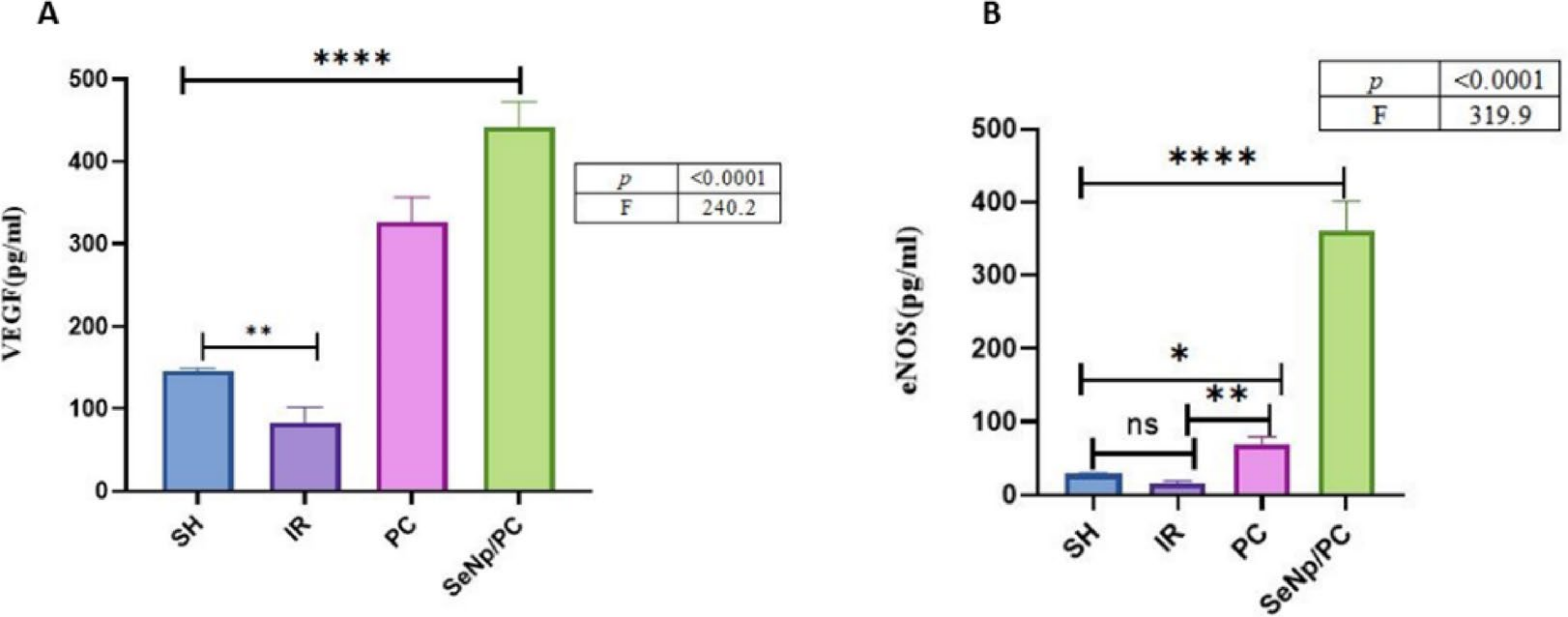
One-way ANOVA followed by Tukey’s post hoc test revealed highly significant differences among groups for both VEGF (F = 240.2, *p* < 0.0001) and eNOS (F = 319.9, *p* < 0.0001). The SeNp/PC group exhibited significantly higher levels of VEGF and eNOS compared to the IR group (****p < 0.0001) and the PC group (**p < 0.01 for VEGF; ****p < 0.0001 for eNOS).

As recorded in (Fig. 5A–B) (Table 4), there was a significant elevation of VEGF & eNOS in SeNp/PC more than PC compared to SH, unlike IR, compared to SH, *p* < 0.0001.

**Table 4:** Concentrations of (a) vascular endothelial growth factor (VEGF) and (b) endothelial nitric oxide synthase (eNOS) in testis homogenates from different study groups. Values are presented as mean ± standard deviation (SD) for each group. Statistical significance was determined using one-way ANOVA, Tukey post hoc test, and GraphPad; differences were considered significant at *p* < 0.05.

Variables	Groups				F test	P value
	SH	IR	PC	SeNp/PC		
VEGF	146.2 ± 2.9	83.31 ± 18.4	325.8 ± 30.7	441.6 ± 30.9	240.2	< 0.0001
eNOS	28.55 ± 1	16.43 ± 2.3	68.80 ± 10.7	361.6 ± 39.3	319.9	< 0.0001

Data are expressed as mean ± SD (n = 7/group). One-way ANOVA followed by Tukey’s post hoc test was used for statistical analysis. Statistical significance is considered when *p* < 0.05.

### Apoptotic and cellular stress markers

**Fig. 6:**
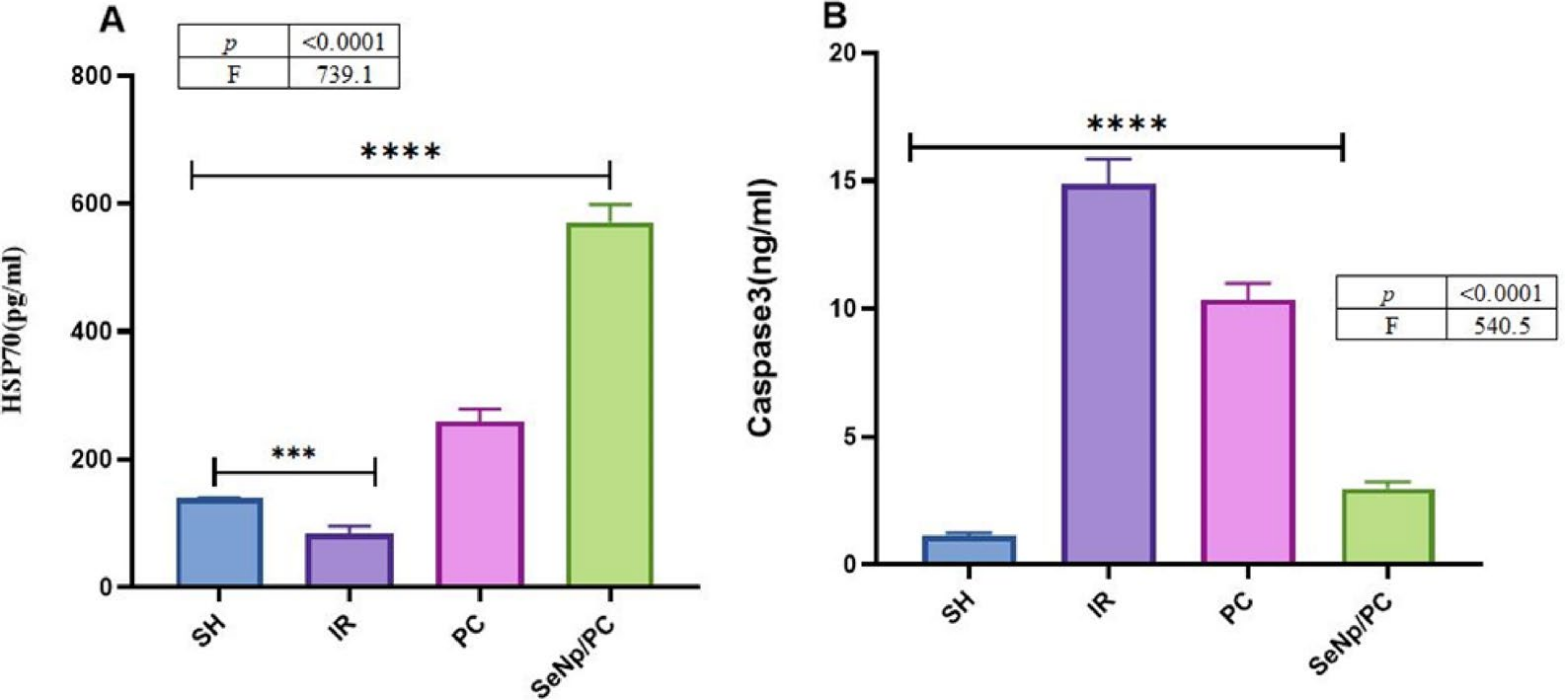
One-way ANOVA revealed highly significant differences among groups for both HSP70 (F = 739.1, *p* < 0.0001) and caspase-3 (F = 540.5, *p* < 0.0001). The SeNp/PC group exhibited a significant increase in HSP70 levels (*p* < 0.001 vs. PC) and a significant decrease in caspase-3 activity (*p* < 0.0001 vs. IR and PC).

HSP70 showed marked upregulation in SeNp/PC than PC alone, compared to IR, while IR was downregulated compared to SH. Caspase3 was markedly elevated in the IR group than SH; however, SeNp/PC showed a highly decreased level compared to PC. while there were a few significant changes between SeNp/PC and SH, *p* < 0.0001. (Fig. 6A–B) (Table 5),

**Table 5:** Concentrations of (a) caspase-3 and (b) heat shock protein 70 (HSP70) in testis homogenates from different study groups. Values are presented as mean ± standard deviation (SD) for each group. Statistical significance was determined using one-way ANOVA, Tukey post hoc test, and GraphPad; differences were considered significant at *p* < 0.05.

Variables	Groups				F test	P value
	SH	IR	PC	SeNp/PC		
Caspase3	1.114 ± 0.1	14.85 ± 0.9	10.33 ± 0.6	2.97 ± 0.2	540.5	< 0.0001
HSP70	139.5 ± 1.1	83.53 ± 12.6	259.6 ± 19.2	571.7 ± 27.5	739.1	< 0.0001

Data are expressed as mean ± SD (n = 7/group). One-way ANOVA followed by Tukey’s post hoc test was used for statistical analysis. Statistical significance is considered when *p* < 0.05.

### Inflammatory and immune response markers

**Fig. 7:**
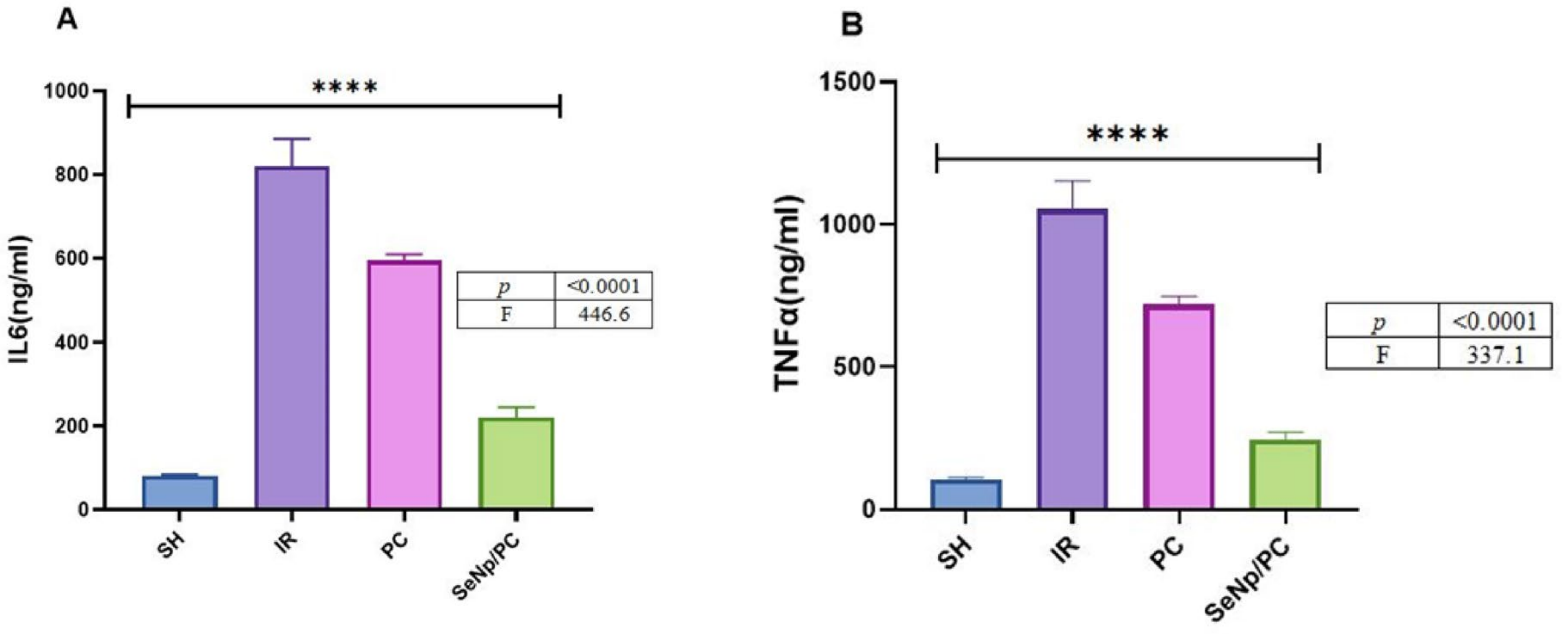
Shows (**A**) Interleukin-6 (IL-6) and (**B**) tumor necrosis factor-alpha (TNF-α) concentrations (ng/ml). Both cytokines were markedly elevated in the I/R group compared to SH controls, while SeNp/PC treatment significantly reduced their levels. Data are expressed as mean ± SD (n = 7per group). Statistical significance was determined by one-way ANOVA followed by Tukey’s test (*p* < 0.0001).

According to the data mentioned in (Fig. 7, Table 6), IR presented an increase in IL6 and TNFα compared to SH. SeNp/PC showed more downregulation than PC alone. SeNp/PC indicated a significantly lower elevation than SH, p<0.0001.

**Table 6:** Concentrations of (a) interleukin-6 (IL-6) and (b) tumor necrosis factor-alpha (TNFα) in testis homogenates from different study groups. Values are expressed as mean ± standard deviation (SD) for each group. Statistical significance was determined using one-way ANOVA, Tukey post hoc test, and GraphPad; differences were considered significant at *p* < 0.0001.

Variables	Groups				F test	*P* value
	SH	IR	PC	SeNp/PC		
IL6	81.17 ± 3.1	820.6 ± 65.2	594.5 ± 15.4	220.3 ± 25.3	446.6	< 0.0001
TNFα	105.6 ± 7.1	1054 ± 98.5	717.9 ± 29.1	246.5 ± 25.7	337.1	< 0.0001

Data are expressed as mean ± SD (n = 7/group). One-way ANOVA followed by Tukey’s post hoc test was used for statistical analysis. Statistical significance is considered when *p* < 0.05.

### Histological evaluation

Histomorphometric evaluation was performed to quantitatively assess testicular architecture. Common morphometric indices include seminiferous tubule diameter, epithelial thickness, germ cell layer count, tubular atrophy index, and Leydig cell area, reflecting spermatogenic and interstitial integrity. In the present study, seminiferous tubule diameter and epithelial thickness were selected due to their reproducibility and strong correlation with spermatogenic activity and tubular health.

**Fig. 8:**
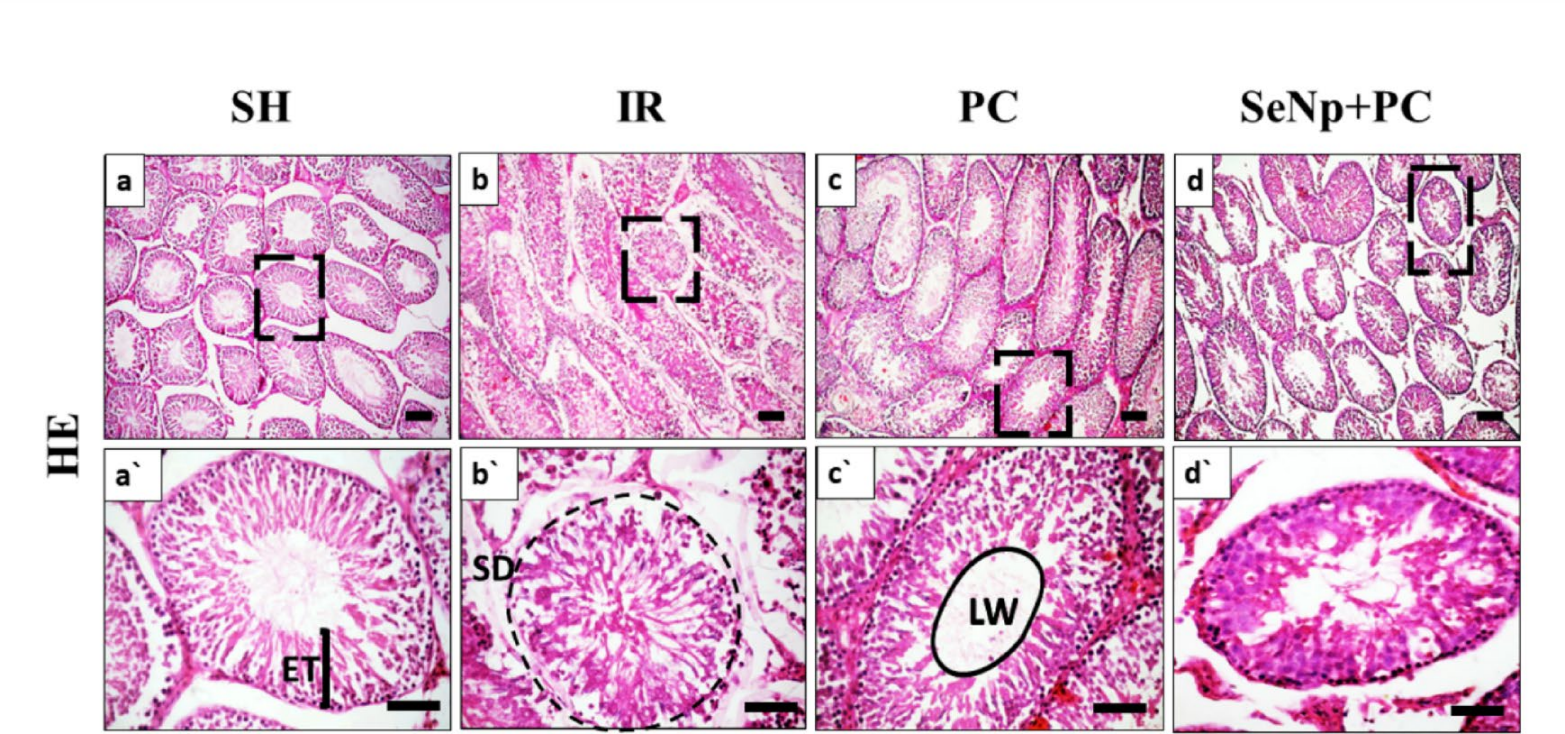
Photomicrograph of HE stained testicular sections of SH group (**a**,**a`**), IR group (**b**,**b`**), PC group (**c**,**c`**), and SeNp + PC group (**d**,**d`**). ET = seminiferous epithelium thickness, SD = seminiferous tubule diameter, LW = luminal width, Scale bars 100 µm (**a**–**d**) and 50 µm (**a`**–**d`**).

HE-stained sections from the SH group revealed normal seminiferous tubules with complete spermatogenesis and preserved tissue architecture (Fig. 8a, a`). In contrast, the ischemia-reperfusion (IR) group displayed degenerated seminiferous tubules with vacuolated epithelium, resulting in a significantly increased epithelial thickness (Figs. 8b,b` & 12a). The PC group exhibited seminiferous tubules with near-normal morphology similar to the SH group; however, the tubules had a notably smaller diameter compared to other groups (Figs. 8c,c` & 12b). Meanwhile, the SeNp+PC group displayed well-preserved seminiferous tubules, with the spermatogenic epithelium reaching up to the spermatozoa stage, yet with a significantly enlarged lumen (Figs. 8d, d `& 12c).

### PAS staining and Johnsen`s scoring

**Fig. 9:**
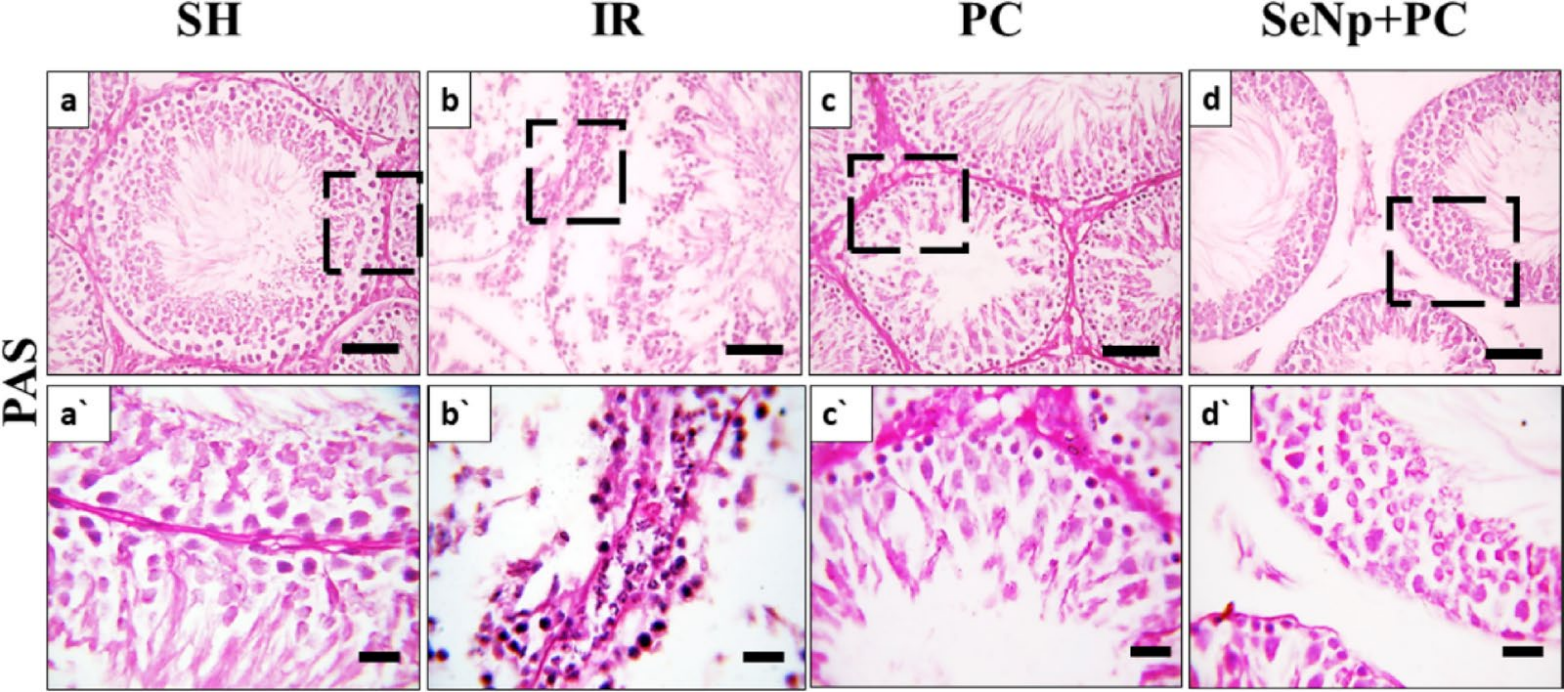
Photomicrograph of PAS-stained testicular of SH group (a, a), IR group (**b**,**b`**), PC group (**c**,**c`**), and SeNp + PC group (**d**,**d`**). Scale bars 50 µm (a-d) and 20 µm (**a`**–**d`**).

PAS-stained sections were utilized to distinguish spermatogenic cell types and assign spermatogenesis scores. Tubules in the SH group typically scored between 8 and 10, indicating complete spermatogenesis with the presence of numerous or few spermatozoa (Figs. 9a, a` & 11). In the IR group, most tubules scored between 2 and 3, containing only spermatogonia or lacking germ cells entirely. Occasionally, tubules scored between 6 and 7, where cells reached the spermatid stage (Figs. 9b, b` & 11). The PC group showed scores ranging from 6 to 9, with some tubules exhibiting nearly complete spermatogenesis and others limited to the spermatid level (Figs. 9 c, c` & 11). The SeNp+PC group demonstrated scores comparable to those of the SH group (Figs. 9 d,d` & 11) *p*<0.01.

#### Immunohistochemical analysis

**Fig. 10:**
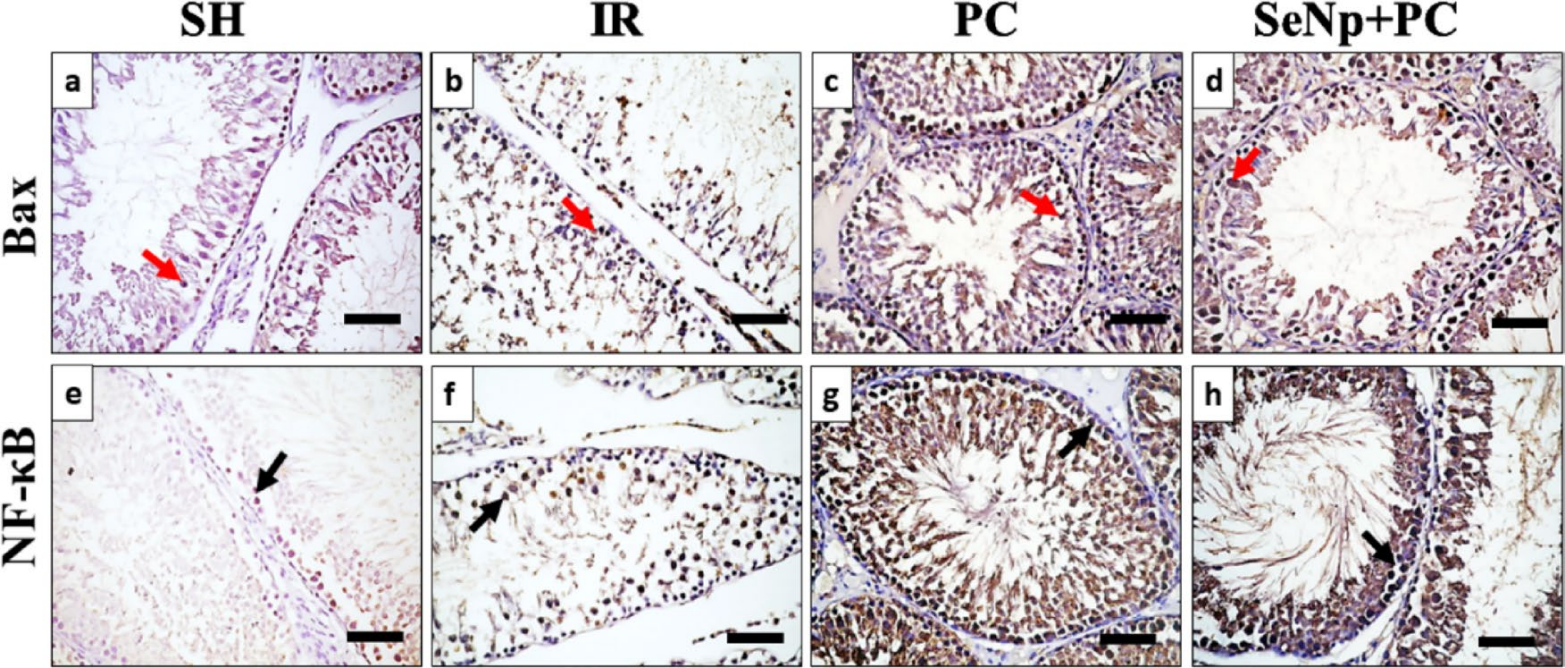
Photomicrograph of immunohistochemically stained testis sections against Bax marker (**a**–**d**) and Nf-κB marker (**e**–**h**) in the tested groups. Red arrows = Bax + cells, black arrows NF-κB + cells. Scale bars 50 µm.

**Fig. 11:**
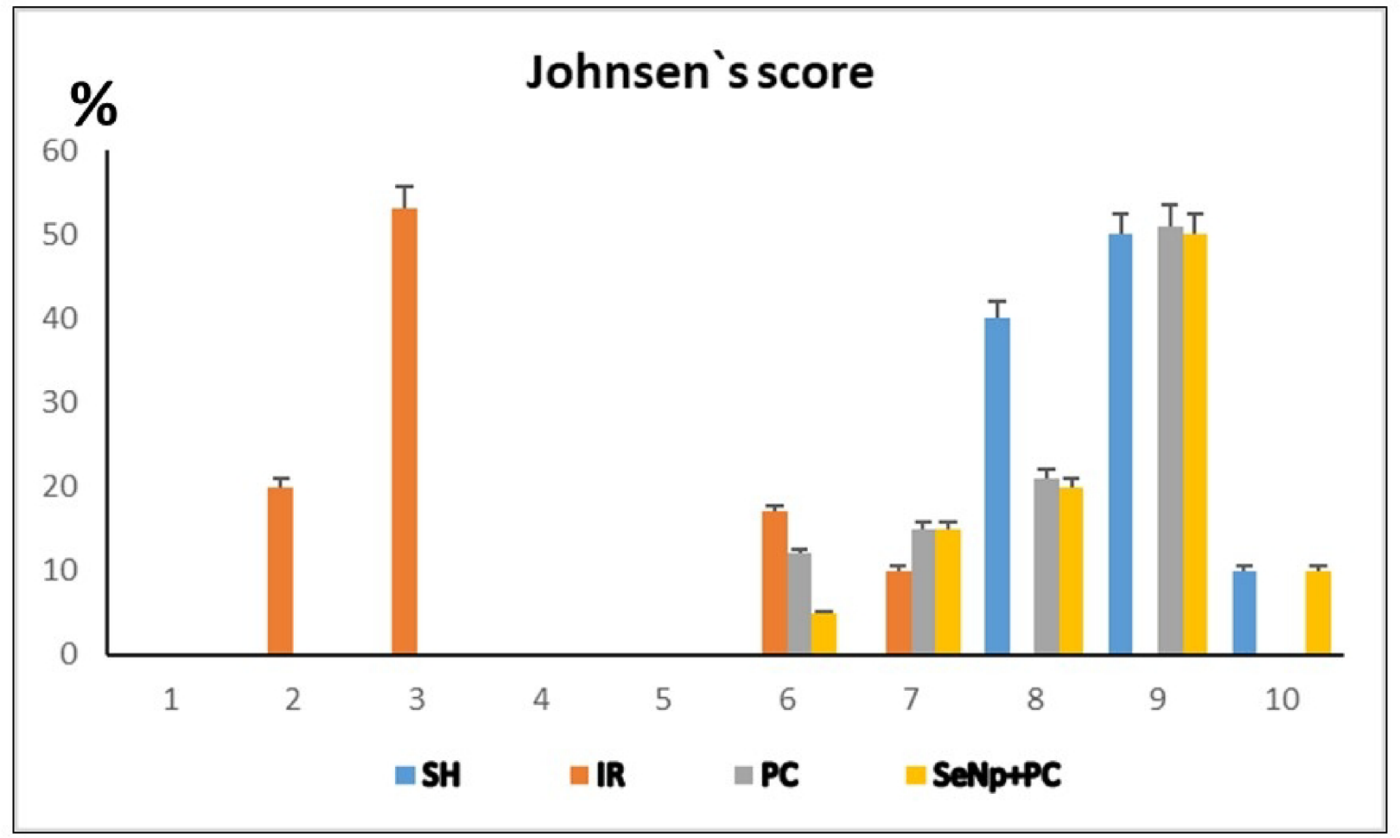
Distribution of Johnsen’s score across the experimental groups.

**Fig. 12:**
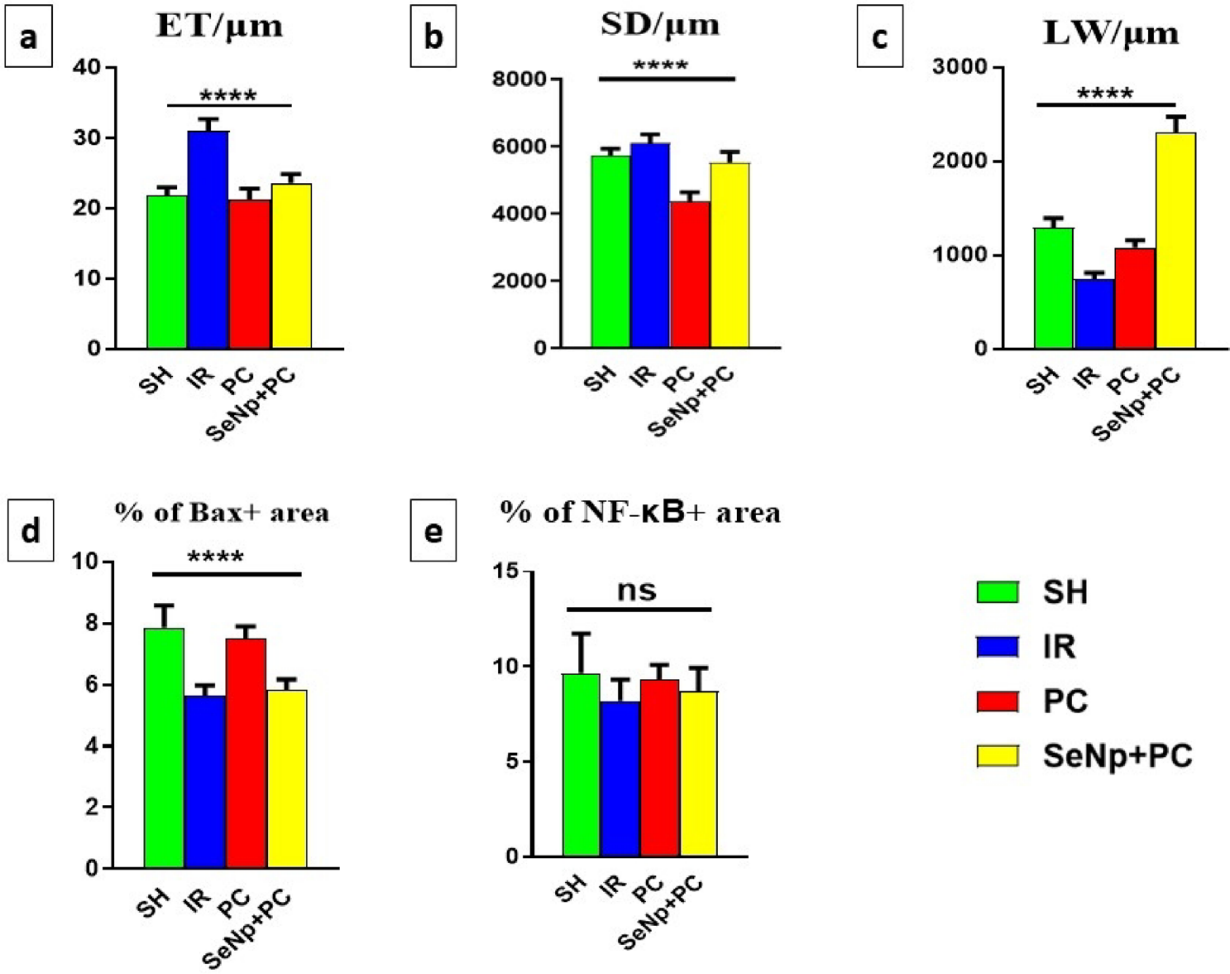
Graphs presenting variable measurements of seminiferous tubules of tested groups, including seminiferous epithelium thickness/µm (**a**), seminiferous tubule diameter/µm (**b**), luminal width/µm (**c**), % of Bax + area (**d**), % of Nf-κB + area (**e**). P-value less than 0.01 (highly significant = ****) and ns = not significant.

Immunohistochemically stained sections against the Bax marker (Fig. 10a–d) and the Nf-κB marker (Fig. 10e–h) were analyzed to measure their percentage of expression, their expression was represented by brown staining in either nuclei or cytoplasm of spermatogenic cells (Fig. 11). The SeNp+PC group exhibited low expression levels of both markers, indicating reduced apoptotic and inflammatory activity (Fig. 12d,e).

The bar chart illustrates the percentage of seminiferous tubules assigned to each Johnsen’s score in the SH, IR, PC, and SeNP + PC groups. The IR group exhibited a marked shift toward low scores (2–3), indicating severe germ cell loss and profound disruption of spermatogenesis following ischemia–reperfusion injury. In contrast, the PC group showed partial improvement, with reduced low-grade scores and higher representation of intermediate grades. Notably, the SeNP + PC group demonstrated the greatest recovery, with a marked increase in high scores (8–10) that closely approximated the normal architecture observed in the SH group. Data are presented as mean ± SD.

## Discussion

The present study demonstrated that (SeNps) in combination with ischemic post-conditioning (PC) provided remarkable protection against testicular (I/R) injury in rats. This protection was evident through biochemical, histological, and immunohistochemical findings, highlighting the synergistic effects of SeNPs and PC in attenuating oxidative stress, inflammation, and apoptosis, while preserving spermatogenesis.

The protective effects of selenium nanoparticles (SeNps) in testicular ischemia–reperfusion models have been well established in several previous studies. Earlier reports demonstrated that SeNPs alone significantly attenuate oxidative stress, reduce lipid peroxidation (MDA), and improve antioxidant enzyme activities (GSH, SOD, CAT) through activation of the Nrf2/HO-1 pathway and modulation of apoptotic markers such as Bcl-2 and caspase-3^32^^,^^33^^,^^34^.

Selenium nanoparticles stimulate the Nrf2/HO-1 pathway, which subsequently upregulates the expression of antioxidant enzymes such as catalase and glutathione peroxidase, as well as other cytoprotective proteins. This activation helps to restore redox balance and mitigate oxidative stress, inflammation, and apoptosis in testicular tissue.^32^.

In parallel, the ERK1/2 signaling pathway, known for its critical role in promoting cell survival and antioxidant defense in various ischemic pre- and post-conditioning models also involved in enhancing cellular resilience and maintaining spermatogenic integrity. The combined activation of these pathways could therefore explain the synergistic protective effects observed in the SeNp plus post-conditioning group^35^.

In addition to the activation of antioxidant pathways, modulation of apoptotic and cytoprotective proteins also contributes to the protective effects observed in the SeNp plus post-conditioning group. Both interventions are known to induce the expression of HSP70, a molecular chaperone that stabilizes intracellular proteins, prevents protein aggregation, and inhibits apoptosis. The upregulation of HSP70 has been linked to improved cellular tolerance and enhanced survival following ischemic stress^33^.

Selecting a single dose of selenium nanoparticles (SeNps), such as 0.5 mg/kg, without conducting a full dose–response validation is based on several practical and scientific considerations drawn from prior research^19^. Previous work has demonstrated that 0.5 mg/kg SeNps can provide significant antioxidant and cytoprotective effects in testicular tissue without overt toxicity, supporting its selection as a reasonable starting point for efficacy studies^36^^,^^37^.

Multiple studies investigating SeNps for testicular protection or antioxidant effects have used doses in the range of 0.1–2 mg/kg, with 0.5 mg/kg falling within this effective and non-toxic window^37^^,^^38^^,^^36^. In addition, higher doses of SeNPs (e.g., 1–2 mg/kg) have been associated with increased risk of toxicity or diminished sperm quality in some animal models, making 0.5 mg/kg a conservative and safer choice for initial investigation^39^.

Testicular I/R injury is well known to disrupt hormonal balance and spermatogenic function. I/R injury leads to increased oxidative stress, inflammation, and apoptosis in testicular tissue, resulting in decreased antioxidant enzyme activity, impaired spermatogenesis, and reduced expression of key genes and proteins necessary for germ cell development and hormone production^40^.

In our study, testosterone, FSH, and LH levels were significantly decreased in the IR group, confirming impaired testicular endocrine activity. Although PC alone partially restored these hormones, SeNp/PC treatment resulted in hormone levels comparable to the SH group, suggesting that SeNps enhance reproductive function, likely through supporting Leydig cell function and reducing oxidative injury. Similar findings were reported by Keshta et al. those who showed that SeNps preserved testicular hormones under toxic stress conditions^41^.

Concerning the evaluation of oxidative, inflammatory, and apoptotic markers, the 24-h post-reperfusion time point provides a comprehensive snapshot of the early molecular events driving tissue damage. These pathways are not independent; rather, they form a tightly interconnected cascade. Oxidative stress acts as the initial upstream trigger, activating pro-inflammatory mediators and subsequently initiating apoptosis in germ and Sertoli cells^42^.

In the present study, the marked reduction in MDA and inflammatory cytokines (TNF-α, IL-6) in the SeNp + PC group advocates effective suppression of the early oxidative insult, which likely diminished downstream inflammatory signaling. This is further supported by the observed decline in caspase-3 expression and the preserved architecture of the seminiferous tubules. Such findings emphasize that the attenuation of oxidative stress is not an isolated effect but a key determinant in limiting the entire inflammatory–apoptotic progression, reflecting a time-dependent protective mechanism.

Moreover, the IR group demonstrated the classical biochemical profile of I/R-induced injury, characterized by elevated MDA and significant depletion of antioxidant defenses (CAT and GSH). Treatment with SeNp + PC not only reduced lipid peroxidation but also restored antioxidant enzyme activity to levels superior to PC alone. This highlights the superior antioxidant competence of SeNps, in agreement with previous reports indicating that SeNps effectively neutralize reactive oxygen species and enhance endogenous antioxidant pathways^43^^,^^44^.

Concerning the Inflammatory mediators, such as TNF-α and IL-6, they were markedly upregulated following IR, reflecting severe inflammatory activation. SeNp/PC administration significantly suppressed these cytokines compared to PC alone, supporting the anti-inflammatory role of SeNps through modulation of NF-κB signaling pathways^45^. This anti-inflammatory effect complements the antioxidant activity, thereby protecting germ cells from secondary damage.

Furthermore, vascular regulatory proteins (VEGF and eNOS) were significantly increased after SeNp/PC treatment, demonstrating enhanced testicular perfusion and vascular repair. VEGF has a well-established role in angiogenesis and Leydig cell function, while eNOS-derived nitric oxide is essential for vascular tone and germ cell survival. These results are in agreement with previous evidence that both SeNPs and ischemic post-conditioning improve endothelial function and vascular integrity^46^^,^^47^.

Apoptotic markers offered additional clarity regarding the protective mechanisms involved. In the IR group, caspase-3 activity was markedly elevated, confirming extensive germ-cell apoptosis. In contrast, SeNp/PC treatment significantly suppressed caspase-3 expression, indicating effective attenuation of the apoptotic cascade. Meanwhile, the upregulation of HSP70a in the SeNp/PC group reflects enhanced cellular resilience against ischemia–reperfusion injury. This observation aligns with the findings of Xia et al., who demonstrated that HSP70 induction plays a crucial role in protecting cells from I/R-mediated damage^48^.

Beyond these effects, both SeNps and post-conditioning appear to concurrently suppress caspase-3 activation while enhancing the expression of the anti-apoptotic protein Bcl-2. Evidence from testicular ischemia models indicates that selenium supplementation reduces caspase-3 and Bax levels and upregulates Bcl-2, thereby shifting the apoptotic balance toward cell survival. These findings support the notion that the combined SeNp and post-conditioning intervention confers protection, at least in part, through the dual mechanism of attenuating pro-apoptotic signaling and reinforcing cytoprotective pathways^32^^,^^49^.

Although the Johnsen’s score did not demonstrate a statistically significant difference among the groups, this outcome is likely related to the relatively short reperfusion interval (24 h) applied in the present experiment. Spermatogenesis is a time-dependent process that requires multiple cycles of germ cell proliferation and differentiation, taking approximately 52 days to be completed in rats^50^; therefore, early morphological changes may not be adequately captured within such a limited recovery period. Despite the absence of statistical significance, both qualitative histological assessment and quantitative morphometric parameters (seminiferous tubular diameter and epithelial height) clearly indicated structural improvement in the SeNP plus post-conditioning group. Accordingly, the non-significant Johnsen’s score should be interpreted as a temporal limitation rather than an indicator of therapeutic inefficacy. Longer follow-up periods are recommended to fully delineate the regenerative potential of SeNPs combined with post-conditioning on spermatogenic restoration.

Evaluation of spermatogenesis using Johnsen’s score further confirmed the biochemical and histological findings. The IR group showed very low scores (2–3), indicating severe disruption of germ cell maturation. Partial recovery was observed in the PC group (scores 6–9), whereas SeNp/PC treatment restored spermatogenesis to nearly normal levels (scores 9–10), comparable to the SH group. These results highlight the strong protective role of SeNPs in preserving germ cell differentiation and spermatogenic progression. Similar protective effects of SeNPs on spermatogenesis under oxidative or toxic stress have been reported previously^43^^,^^44^.

Histopathological and immunohistochemical assessments reinforced these biochemical findings. IR caused severe degeneration of seminiferous tubules and markedly reduced Johnsen’s scores. PC partially restored spermatogenesis, while SeNp/PC nearly normalized testicular histology with complete germ cell maturation up to spermatozoa. Additionally, immunostaining showed reduced expression of Bax and NF-κB in the SeNp/PC group, confirming decreased apoptosis and inflammation at the tissue level. These outcomes corroborate prior reports that SeNPs reduce apoptotic signaling and maintain testicular structure under oxidative stress conditions^51^.

The histological enhancements found after SeNp plus post-conditioning therapy were strongly related to the favourable biochemical assessment, showing a robust structural reflection of molecular modulation. The suppression of oxidative stress and inflammatory signalling was followed by the preservation of seminiferous tubule architecture and germinal epithelium integrity, indicating a mechanistic relationship between redox equilibrium and tissue survival. Furthermore, reduced apoptotic activity was associated with improved spermatogenic organization, indicating that pharmacological modulation of oxidative and apoptotic pathways leads directly to histological protection and functional recovery after testicular ischemia–reperfusion damage.The combination of SeNps and PC offers mechanistic synergy: SeNps directly scavenge ROS and restore antioxidant enzyme activity, while PC primes the tissue to better withstand oxidative insults. This dual approach not only reduces oxidative stress more effectively than either intervention alone but also addresses both the immediate and sustained phases of reperfusion injury, potentially leading to superior preservation of testicular structure and function^52^.

### Translational implications and study limitations

From a translational standpoint, the combined application of selenium nanoparticles and ischemic post-conditioning represents a potentially valuable therapeutic approach for minimizing testicular injury following torsion–detorsion events. Nevertheless, several challenges must be addressed before this strategy can advance toward clinical utilization.

One important consideration relates to the interpretation of hormonal findings particularly FSH and LH which are regulated by the hypothalamic–pituitary–gonadal (HPG) axis through a well-established negative feedback mechanism. Increases or decreases in serum testosterone directly modulate pituitary secretion of FSH and LH; therefore, isolated changes in gonadotropins cannot be attributed solely to improved testicular function without excluding upstream pituitary involvement. To confirm that hormonal restoration originates from testicular recovery rather than altered central regulation, additional analyses—such as GnRH stimulation testing, pituitary morphometry, or expression of steroidogenic enzymes (e.g., StAR, CYP11A1, 3β-HSD)—would provide complementary mechanistic evidence.

The optimal human administration route for SeNps remains undefined, and systemic delivery may differ from local administration in bioavailability and tissue targeting. Moreover, despite their favorable biocompatibility at low doses, uncertainties remain regarding long-term safety, potential nanoparticle accumulation, and interactions with reproductive tissues.

The current experimental model also reflects an acute 24-h assessment window, limiting conclusions about sustained structural recovery and long-term spermatogenic performance. Thus, while the present findings provide valuable mechanistic insight, extended preclinical studies including chronic exposure models and detailed pharmacokinetic profiling are required to establish both the feasibility and safety of SeNp-based reproductive therapies.

Future investigations should lengthen the follow-up period to evaluate long-term fertility outcomes and spermatogenic restoration. In addition, a larger panel of inflammatory and apoptotic markers should be assessed to offer a broader understanding of the underlying biological processes. Incorporating Doppler ultrasonography could also enhance characterization of testicular hemodynamics during ischemia and reperfusion, offering a more precise evaluation of vascular and microcirculatory responses. Furthermore, SeNp-only groups should be included to allow for better separation between independent and synergistic effects, as well as confirmation of possible synergistic interactions.

## Conclusion

SeNPs combined with post-conditioning represent a promising therapeutic approach to mitigate ischemia–reperfusion injury following testicular torsion. Their synergistic actions provide a strong rationale for further preclinical investigations and potential translation into clinical settings.

## Data Availability

All data generated or analyzed during this study are included in this published article and its supplementary materials, including detailed results presented in the attached supplementary graphic abstract.

## Abbreviations

AMP: AMP-activated protein kinase
ANOVA: Analysis of variance
Bax: Bcl-2-associated X protein
Bcl-2: B-cell lymphoma-2
BSA: Bovine serum albumin
CAT: Catalase
CM: Carboxymethyl cellulose
DAB: 3,3′-Diaminobenzidine
DLS: Dynamic light scattering
DNA: Deoxyribonucleic acid
ELISA: Enzyme-linked immunosorbent assay
ERK1/2: Extracellular signal-regulated kinases 1 and 2
ET: Epithelial thickness
FSH: Follicle-stimulating hormone
GnRH: Gonadotropin-releasing hormone
GPx: Glutathione peroxidase
GSH: Reduced glutathione
HB: Hemoglobin
HE/H&E: Hematoxylin and eosin
HIF-1α: Hypoxia-inducible factor-1 alpha
HPG axis: Hypothalamic–pituitary–gonadal axis
HRP: Horseradish peroxidase
HSP70: Heat shock protein 70
HCT (PCV): Hematocrit/Packed cell volume
IL-6: Interleukin-6
IPostC/PC: Ischemic post-conditioning
IR: Ischemia–reperfusion
i.p: Intraperitoneal
I/R: Ischemia–reperfusion
LH: Luteinizing hormone
LW: Luminal width
MDA: Malondialdehyde
MCV: Mean corpuscular volume
MCH: Mean corpuscular hemoglobin
MCHC: Mean corpuscular hemoglobin concentration
NF-κB: Nuclear factor kappa-light-chain-enhancer of activated B cells
NO: Nitric oxide
NOS: Nitric oxide synthase
eNOS/NOS3: Endothelial nitric oxide synthase
PAS: Periodic acid–Schiff
PBS: Phosphate-buffered saline
PDI: Polydispersity index
PKB-Akt: Protein kinase B
ROS: Reactive oxygen species
RBCs: Red blood cells
RIPostC: Remote ischemic post-conditioning
PC: Post-conditioning
SOD: Superoxide dismutase
SD: Seminiferous tubule diameter/Standard deviation (defined in text context)
SeNps: Selenium nanoparticles
SH: Sham group
StAR: Steroidogenic acute regulatory protein
TEM: Transmission electron microscopy
TNF-α: Tumor necrosis factor-alpha
VEGF: Vascular endothelial growth factor
